# The Role of Reactive Oxygen Species in the Rheumatoid Arthritis-Associated Synovial Microenvironment

**DOI:** 10.3390/antiox11061153

**Published:** 2022-06-13

**Authors:** Xing Wang, Danping Fan, Xiaoxue Cao, Qinbin Ye, Qiong Wang, Mengxiao Zhang, Cheng Xiao

**Affiliations:** 1 School of Clinical Medicine, China-Japan Friendship Hospital, Beijing University of Chinese Medicine, Beijing 100029, China; 20210941326@bucm.edu.cn (X.W.); 20190931087@bucm.edu.cn (Q.Y.); 20200941260@bucm.edu.cn (Q.W.); 2 Institute of Clinical Medicine, China-Japan Friendship Hospital, Beijing 100029, China; dpfan@student.pumc.edu.cn (D.F.); snowcxx93@student.pumc.edu.cn (X.C.); 15703290131@163.com (M.Z.); 3 Graduate School of Peking Union Medical College, Chinese Academy of Medical Sciences and Peking Union Medical College, Beijing 100193, China; 4 Department of Emergency, China-Japan Friendship Hospital, Beijing 100029, China

**Keywords:** rheumatoid arthritis, reactive oxygen species, synovium, synovium microenvironment

## Abstract

Rheumatoid arthritis (RA) is an inflammatory disease that begins with a loss of tolerance to modified self-antigens and immune system abnormalities, eventually leading to synovitis and bone and cartilage degradation. Reactive oxygen species (ROS) are commonly used as destructive or modifying agents of cellular components or they act as signaling molecules in the immune system. During the development of RA, a hypoxic and inflammatory situation in the synovium maintains ROS generation, which can be sustained by increased DNA damage and malfunctioning mitochondria in a feedback loop. Oxidative stress caused by abundant ROS production has also been shown to be associated with synovitis in RA. The goal of this review is to examine the functions of ROS and related molecular mechanisms in diverse cells in the synovial microenvironment of RA. The strategies relying on regulating ROS to treat RA are also reviewed.

## 1. Introduction

Rheumatoid arthritis (RA) is a synovial illness characterized by systemic immune dysregulation, which is caused by a complex interplay of hereditary and environmental factors [1,2]. The progression of RA from preclinical disease to evident synovitis is currently thought to be driven by abnormal adaptive immunity, including mucosal immune responses that began in previous years [3]. Furthermore, infiltrate immune cells in RA synovial tissue interact with tissue-resident cells, resulting in qualitative alterations in cell phenotypes, boosting inflammation and tissue death and hindering inflammation elimination [4]. When leukocytes migrate to joints where simultaneous innate and adaptive responses develop [5], they activate a powerful burst of oxidation under the stimulation of inflammatory factors. During this period, a large amount of oxygen is consumed and converted into a variety of free radicals and non-free-radical oxidation, forming a local environment of highly reactive oxygen, which plays a very important role in the occurrence and persistence of RA [6].

Reactive oxygen species (ROS) are molecules with at least one oxygen atom and one or more unpaired electrons and can exist independently [7]. This group includes oxygen and nitrogen radicals. Under physiological conditions, they are formed in small amounts in cells and participate in oxygen respiration. In addition, ROS are primarily signaling molecules and can induce cell differentiation and apoptosis. There is a balance between the formation and clearance of free radicals in the body [8]. When ROS are over-generated and/or the antioxidant system of the body is damaged, ROS and its related metabolites accumulate excessively, thus causing tissue damage, which is called oxidative stress [9]. Currently, the evidence strongly suggests that oxidative stress plays a role in the pathogenesis of RA [10,11]. According to one study, there is a robust link between oxidative stress levels and synovial fluid oxidative damage markers [12]. In the blood of RA patients, the majority of ROS had strong positive relationships with clinical and biochemical indicators [13]. As a result, ROS could be useful indicators for tracking disease development, since oxidative stress plays a key role in RA pathogenesis. 

There is a large amount of ROS produced by phagocytes, recruited immune cells and proliferating synovial stromal cells in the RA synovitis microenvironment, but the complex interactions between ROS and these cells remain unclear. Therefore, the primary aim of this paper is to review the role and molecular mechanisms of ROS in various cells in the RA synovial microenvironment and summarize the therapeutic intervention of ROS to restore the redox balance in RA patients, providing a new research direction for treating RA.

## 2. The Production of ROS in Synovitis

**Figure 1:**
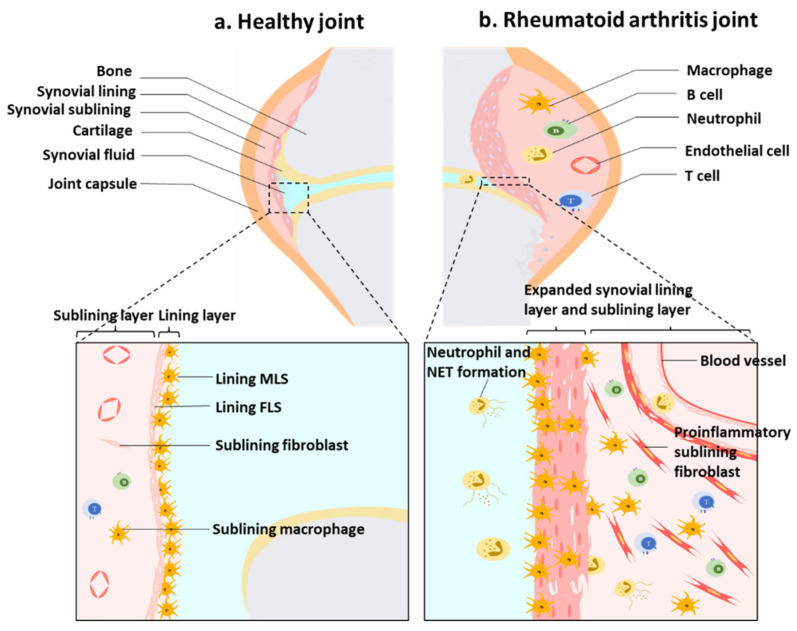
The synovial structure in healthy individuals and in individuals with rheumatoid arthritis (RA). (**a**) In the healthy joint, the synovial intimal lining consists of a thin cell layer of fibroblast synovial cells (FLSs) and macrophage-like synovial cells (MLSs) that together form a semi-permeable protective barrier. The sublining layer contains interstitial macrophages and fibroblasts, as well as blood vessels. (**b**) In RA, the macrophage barrier is lost and there is pathological expansion and remodeling of the synovial lining layer and sublining layer leading to synovial hyperplasia. The sublining of the synovium is heavily infiltrated by immune cells and undergoes neovascularization.

The synovial tissue is the main site of RA disease activity, and there is location-specific and stage-specific cell diversity. The synovial membrane can be divided into two distinct regions at the gross histological level: the inner lining and the sublining [4]. In healthy joints, the lining is only 1–3 layers thick and consists of macrophage-like synovial cells (MLSs) and fibroblast synovial cells (FLSs). The sublining is composed of interstitial macrophages and fibroblasts, including blood vessels, lymphatics and nerves [4,14]. Unlike recruited monocyte-derived pro-inflammatory macrophages, synovial macrophages (SMs) form a tightly connected immune barrier at the synovial lining synovial, isolating the joint cavity and limiting the inflammatory response [15]. Other immune cells, including lymphocytes, mast cells and dendritic cells, are rare in normal synovium and can only be found in the perivascular portions of the sublining layer [16]. In RA, infiltrating immune cells participate in the settlement of macrophages and the proliferation of fibrocytes in the tissue, and the lining can be 10 to 20 cells thick, leading to synovial hyperplasia [17]. In addition, the sublining of the synovium is heavily infiltrated by immune cells and undergoes neovascularization (Figure 1). Arthroscopic observations of RA joints show swollen, edematous intima synovium tissue with red hyperemia and often villi formation [4].

Hypoxia and inflammation are characteristic of RA synovitis. Multiple previous studies have shown that synovitis in RA is characterized by severe hypoxia. Ursula et al. developed a method to measure oxygen tension (pO_2_) in synovial tissue directly using an arthroscope and a combined pO_2_ and temperature probe, and showed severe hypoxia in inflammatory synovial joints (median synovial oxygen levels of 3.2% [range 0.46–7%]), which has been thoroughly confirmed [18]. In addition, the pO_2_ levels in synovial tissue were found to be negatively correlated with the number of CD3^+^ and CD68^+^ cells in the synovial sublining layer in vivo [18]. In another study, it was found that mitochondrial DNA mutations, mitochondrial membrane potential, mitochondrial mass, ROS and glycolysis activity increased in primary RA synovial fibroblasts (RASFs) under hypoxia, while mitochondrial respiration and ATP activity decreased [19]. In contrast, mitochondrial dysfunction induces an inflammatory response and significantly amplifies it in normal human synovial cells. Mitochondria undergo oxidative phosphorylation (OXPHOS) to oxidize nutrients, which in turn form adenosine triphosphate (ATP), which inevitably produces the highly reactive and toxic by-product ROS [20]. Increased oxidative stress contributes to somatic mitochondrial DNA (mtDNA) alterations because the mitochondrial genome is especially sensitive to mutagenesis [21,22]. Studies found mtDNA mutations in synovial tissue and somatic cells of RA patients and increased mitochondrial mutagenesis associated with reduced pO_2_ in synovial membranes, which induced a pro-inflammatory mitochondrial phenotype [21,22,23]. Therefore, hypoxia and oxidative stress may be important drivers of the inflammatory process in arthritic joints [24]. 

ROS are created not only during OXPHOS, but also by NADPH oxidase complexes (NOXs) at significantly greater levels. NOX_2_, the most well-known and powerful of these complexes, is mostly expressed in phagocytic cells, such as neutrophils, monocytes and macrophages, as well as dendritic cells (DCs), and it is the most important ROS-producing complex in mammals [25]. The discovery of a NOX family poses new possibilities for studying the various functions of the ROS generation by genetically modifying these sources [26]. Typically, ROS can be produced by activated macrophages and chondrocytes in the synovial membrane, as well as by activated neutrophils in the synovial capsule. The superoxide anion (O_2_^•−^), singlet oxygen (^1^O_2_), hydroxyl radical (^•^OH), hydrogen peroxide (H_2_O_2_) and hypochlorous acid (HOCl) can damage DNA, proteins, lipids and many other molecules, and are the most prominent ROS implicated in inflammatory insults to tissues [27]. In addition, reactive nitrogen species (RNS), such as peroxynitrite (ONOO^−^) and nitric oxide (NO^•^), are also thought to be mediators of synovial tissue and joint destruction in RA patients [28,29]. ROS production, lipid peroxidation, protein oxidation and DNA damage were increased in the peripheral blood of RA patients, and antioxidant levels, such as the iron reduction ability and DPPH radical quenching ability, were significantly decreased [13,30]. 

4-Hydroxy-2-nonenal (4-HNE) is a significant aldehyde produced during lipid peroxidation from superoxide. It reacts with proteins, DNA and phospholipids, and functions as an oxidative stress inducer and mediator. Monika et al. reported that increased 4-HNE levels were associated with higher clinical disease activity scores and angiogenic marker expression in the synovial tissue of RA patients [31]. In RASF, 4-HNE stimulates mtDNA mutation and ROS increase, and activates proangiogenic responses that subsequently alter human umbilical vein endothelial cell (HUVEC) invasion and migration, angiogenic tube formation and proangiogenic mediator release [32]. Most notably, a recent study showed that anti-tumor necrosis factor (TNF)-α treatment significantly decreased lipid peroxidation, indicating that it is a crucial event in arthritis [33]. 8-Hydroxyl-2′-deoxyguanosine (8-oxodG), a marker of DNA oxidative damage, has been found in the synovial fluid (SF) and blood of RA patients and is associated with rheumatoid factors in RA patients [34]. Thus, epitope transmission in RA may be aided by neoangiogenic determinants produced by ROS and RNS [35]. In addition, elevated markers of oxidative stress, such as glycosylation, oxidation, nitrifying proteins and amino acids, were detected in the synovial fluid of RA patients. These acids can be used as biomarkers to diagnose and classify arthritic disorders early on [36,37]. 

Iron is found in the synovial membrane of lesions and is known to be a catalyst for H_2_O_2_ production of ^•^OH via the Fenton reaction [38]. The degenerative process of RA development may be aided by lipid peroxidation-related ferroptosis [39,40]. Notably, sulfasalazine indirectly increases ferric iron levels, triggering the Fenton reaction, which produces excess lipid ROS and causes ferroptosis [41]. Ferroptosis could be used as a therapeutic target in the treatment of RA.

Moreover, hyperglycemia and oxidative stress cause IgG to be glycated. In RA, auto-antibodies to advanced glycation end-product (AGE)-IgG and anti-AGE-IgG IgM have been discovered, contributing to the pathophysiology of the disease [42]. In addition, type-II collagen that has been post translationally changed by reactive oxygen species (ROS-CII) found in the SF is an auto-antigen in RA, which has the potential to become a diagnostic biomarker of RA [43]. Advanced oxidation protein products observed in RA patients’ synovial fluid are implicated in a variety of inflammatory processes involving NOX-dependent ROS and are used as RA oxidative stress markers [12,44]. 

Furthermore, transient receptor potential vanilloid 4 (TRPV4) is a Ca^2+^-permeable channel that relates to ATP release and is involved in the development of RA. The hypotonicity of synovial fluid elicits Ca^2+^ entry via the TRPV4 channel, which increases ATP release and ROS generation, hence exacerbating synovial fibroblast proliferation [45]. TRPV4 has also been demonstrated to mediate the formation of ROS in response to pro-inflammatory stimuli, which has been linked to the activation and differentiation of innate immune cells, including neutrophils and macrophages [46]. 

## 3. ROS and Synovium Stromal Cells

### 3.1. The Role of ROS in FLSs

The former compartment of the synovium contains highly specialized mesenchymal cells known as FLSs [47]. FLSs regulate the extracellular matrix and synovial fluid composition, keeping cartilage surfaces lubricated and nourished [48]. Notably, FLSs have distinct aggressive behavior in RA that have a role in disease causation and development. Part of the FLS phenotype in RA patients is a passive reaction to the in vivo inflammatory environment, with partly imprinted features that persist in cell culture in vitro, and the latter, at least partly, through the epigenetic modification of the genome [49,50]. 

#### 3.1.1. The Role of ROS-Induced Gene Mutations in FLSs

**Figure 2:**
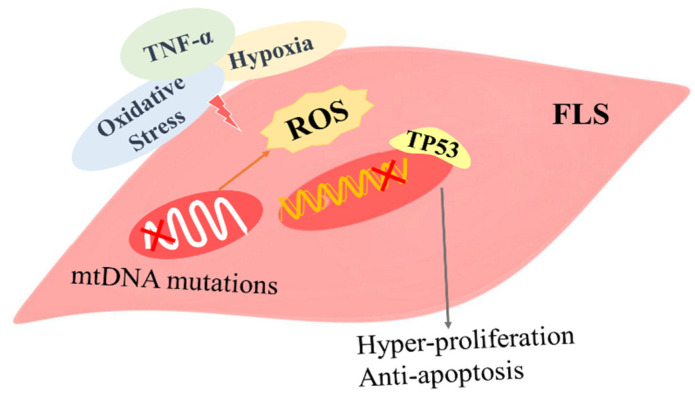
Role of reactive oxygen species (ROS)-induced gene mutations in fibroblast synovial cells. The synovial microenvironment of RA with hypoxia, high ROS and high inflammatory factors may lead to genetic imprinting of FLS by somatic and mitochondrial DNA mutations (as shown in the Red Cross), which lead to FLS proliferation and anti-apoptosis.

ROS and reactive nitrogen species are known to be produced by the synovial tissues of RA and may induce mutations in key genes. According to Sang-Heon and colleagues, exposing RA FLSs to a nitric oxide donor causes an imbalance in the expression of two DNA repair genes, MutS Homolog 6 (MSH6) and MSH3, which could explain their enhanced microsatellite instability [51]. TP53 mutations encode the cellular tumor antigen p53, and they have been found in RA during several studies [52]. The genome-maintaining protein p53, widely known as the “guardian of the genome,” inhibits cells with damaged DNA from proliferating [53]. Transition missense mutations are the most common type of p53 mutation (approximately 80%), and they are caused by nitric oxide deamination [54]. Because mutant p53 has a longer half-life, its expression in the synovium is increased in RA [55] and FLSs may be protected from apoptosis by dominant-negative p53 mutations, which may contribute to FLS invasiveness [56] (Figure 2).

Other genes, such as mtDNA in RA FLS, have also been shown to contain functional mutations [57]. In vitro, TNF-α stimulation resulted in RASF mitochondrial dysfunction and mtDNA mutation, and there was a strong link between mtDNA mutation frequency and the levels of inflammation and the pro-inflammatory cytokines TNF-α and IFN-γ (Figure 2). In patients with a DAS score < 3.2, the mtDNA mutation frequency was significantly reduced after TNFi treatment and correlated with clinical and microscopic measurements of disease [22,57]. Furthermore, exposing immortalized RA cells to 1% hypoxia increased the number of mtDNA mutations in vitro, which could be avoided with antioxidant therapy [21]. Ex vivo cultured FLSs and synovial tissue from RA patients show shorter mitochondria and increased production of the mitochondrial fission GTPase dynamin 1-like protein (DNM1 L) [58,59]. In a collagen-induced arthritis (CIA) mouse model, inhibiting mitochondrial fission with a GTPase inhibitor decreased synovial tissue ROS levels, lowered disease severity and suppressed the production of inflammatory and destructive mediators [59]. These findings support the intricate interaction between oxidative damage, somatic mutations and inflammatory pathways during the pathogenesis of inflammatory arthritis.

M^6^A (N^6^-methyladenosine) is the most common epigenetic regulator in eukaryotes, where the dysregulation of m^6^A has been linked to human diseases, including RA [60]. Our previous review showed that m^6^A modifications are crucial in various RA-associated cells, such as T cells, macrophages [60] and FLS cells [61], implying that m^6^A modifications provide evidence for RA pathogenesis. In living organisms, oxidative stress produces around 10^4^ DNA damages per cell every day [62]. As a result, quick and accurate oxidative damage repair is required to protect genomic integrity and prevent lesions. A recent study showed that the post-translational modification of the RNA m^6^A demethylase ALKBH5 induced the expression of DNA damage-repair-related genes by increasing overall m^6^A levels in response to ROS-induced DNA damage, while the inhibition of RNA m^6^A methylation enhanced ROS-induced DNA damage and apoptosis [63]. This study suggests that RNA m^6^A methylation may play an important role in protecting the genomic integrity of cells in response to ROS. FLS, as a key effector cell in RA capable of promoting immune and inflammatory responses and displaying cancer-like features [48], could be used in future studies to investigate the important role of m^6^A modification and ROS in RA.

#### 3.1.2. The Role of ROS-Related Autophagy and Apoptosis in FLSs

It is well known that FLS apoptosis in RA is downregulated, and progressive cartilage and bone destruction is caused by the invasive phenotype of FLS [64,65]. In addition, the levels of the autophagy-related molecules Beclin-1,Atg5 and LC3-II in the synovial tissues of RA patients are increased [66], and there is a negative correlation with apoptosis [67]. Because ROS have been linked to autophagosome production through the regulation of Atg4 function [68], FLSs may protect themselves against apoptosis through the stimulation of autophagy by increasing the ROS level [69]. The activation of autophagy can ensure the right balance of oxidizing species levels in cells by recycling damaged mitochondria that produce ROS [70]. Redox-active trace metals (RATMs) stimulated RA FLSs expressing the TLR4 receptor, increased intracellular ROS, promoted the release of high-mobility group Box 1 (HMGB1) and autophagy and had an antagonistic effect on cell apoptosis [71]. In addition, DNM1L upregulation can also promote FLS survival by boosting the expression of autophagy LC3B and ROS production, inhibiting apoptosis and stimulating inflammation by regulating AKT signaling [59]. Therefore, several pathways stimulate FLS autophagy, counteract apoptosis and boost the cell’s ability to proliferate. Basin, a traditional Chinese medicine known as *Caesalpinia sappan* L., leads to increased ROS and autophagy levels in RAFLs and limits inflammatory responses by inhibiting the NF-KB pathway [72]. However, resveratrol, a bioactive phenolic substance, was discovered to downregulate autophagy-related proteins in H_2_O_2_-treated FLS cells, and enhance ROS generation and Ca^2+^ release, resulting in FLS cell death [73]. As a result, autophagy triggered by ROS could be a beneficial or detrimental cellular response to oxidative stress [69]. It is suggested that there is a complex interaction between ROS and autophagy, which requires further study.

Autophagy is a cell-survival process, whereas apoptosis is a deliberate cell-killing process, and mitochondria-mediated apoptosis is the endogenous pathway of cell apoptosis [74]. Recent studies have shown that exposure to many chemical drugs or active components of traditional Chinese medicine can induce the accumulation of mitochondrial reactive oxygen species (mtROS) in FLS cells, and subsequent oxidative stress in mitochondria can lead to the rapid depolarization of the internal mitochondrial transmembrane potential (MTP) and destruction of oxidative phosphorylation (OXPHOS). In addition, it induced the apoptosis of FLSs by destroying the mitochondrial membrane and function [74]. Through the formation of pores in the outer membrane of mitochondria, the mitochondrial apoptosis pathway increases the permeability of the outer mitochondrial membrane, so that cytochrome C (Cyt-C) and other apoptosis-inducing factors are continuously released into the cytoplasm. Cyt-C binds to caspase 9 to form apoptotic bodies, which activate caspase 3 and lead to cell apoptosis [75,76]. Notably, mtROS accumulation precedes (MCMP) damage, nuclear aggregation and apoptotic body formation, which is a key difference between necrotic apoptosis and mitochondrial pathway apoptosis [77,78]. 

In conclusion, the intracellular and extracellular ROS levels of FLS play an important role in regulating the anti-apoptosis and pro-proliferation effects of FLSs.

### 3.2. The Role of ROS in MLS

Multiple inflammatory cells infiltrate the synovium throughout time, which is a hallmark of RA. The degree of synovitis is correlated with disease activity and is directly connected to monocyte recruitment, resulting in an increase in the total number of synovial macrophages [79]. Macrophages react to pathogen-associated molecular patterns (PAMPs) and damage-associated molecular patterns (DAMPs) through a variety of pattern recognition receptors (PRRs), and form immune complexes by combining Fc receptors with autoantibodies, thus driving the pro-inflammatory effect of macrophages [80]. When activated, macrophages produce not only a variety of pro-inflammatory cytokines, but also nitric oxide (NO) and superoxide, which are highly destructive to microorganisms and the surrounding tissues [81]. Moreover, ROS derived from NADPH oxidase (NOX2) in macrophages play an important role in regulating the activation of adjacent T cells through antigen presentation [82].Conversely, the increase in cytokines and oxidized lipids in the synovitis microenvironment can also affect the activation, polarization and apoptosis of macrophages [79]. Exposure to the synovial fluid of RA rich in oxosterol further enhances macrophage sensitivity to these ligands, thereby increasing the secretion of pro-inflammatory factors [83]. 

Macrophage polarization refers to the ability to display different activation states in response to the extracellular environment, specifically the so-called pro-inflammatory M1 phenotype and anti-inflammatory M2 phenotype [84]. M1-type macrophages are considered to have a pro-inflammatory phenotype, producing ROS and NO through NADPH oxidase and nitric oxide synthase and exerting a strong cytotoxic capacity [85]. In contrast, M2-type macrophage activation plays a role in inflammation reduction and tissue remodeling, leading to a decrease in ROS and NO production by stimulating increased Arginase-1 activity [85]. In RA blood and synovial tissue, macrophages showed an imbalance in M1 and M2 subgroups. It was reported that 68% of macrophages in the synovial fluid of RA patients are M1 macrophages [86]. This imbalance in macrophage homeostasis is a major contributor to pro-inflammatory mediators in RA, leading to the continuous activation of immune cells and stromal cells and accelerated tissue remodeling in the synovial microenvironment.

**Figure 3:**
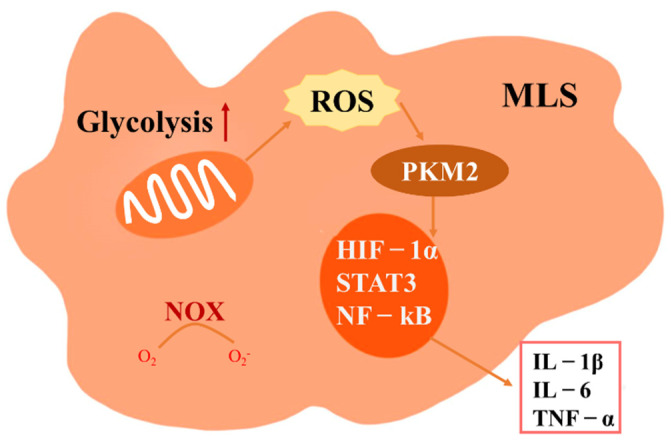
The role of ROS in macrophage-like synovial cells (MLSs). Under conditions, such as the inflammation and hypoxia that occur in RA joints, the MLSs switch to a high-load glycolytic metabolism, resulting in ROS leaking from the electron transport chain and the activation of pyruvate kinase M2 (PKM2), which in turn activates key downstream transcription factors, such as STAT3, NF-ΚB and hypoxia-inducible factor-1 α (HIF-1α), to drive TNF-α, IL-1β and IL-6 production.

In RA patients, transcriptome profiling of FLSs and MLSs isolated from synovial tissues confirmed that MLSs are macrophages that have strong inflammatory tendencies [87]. Treating with anti-TNF drugs can rapidly and significantly improve the clinical symptoms of patients with tissue-infiltrating MRP^+^ macrophages, suggesting that targeting infiltrating macrophages plays an important role in human autoimmune synovitis [88]. In RA, macrophages in the joint are continuously influenced by the synovial inflammatory environment. Studies have shown that ROS play an important pathogenic role in the abnormal metabolism of macrophages during the chronic inflammation of RA joints. Due to the low oxygen, high ROS, lactic acid and succinic acid in RA synovium, activated macrophages undergo metabolic transformation from oxidative phosphorylation to aerobic glycolysis. The balance between the decomposition of metabolites during the TCA cycle and the ability of the electron transport chain to absorb and assimilate electrons is broken down, and excessive pyruvate in mitochondria increases ROS leakage in the electron transport chain (ETC), leading to post-translational modifications of the cytoplasmic enzyme pyruvate kinase M2 (PKM2). Activated dimer PKM2 activates key transcription factors, such as hypoxia-inducible factor-1 α (HIF-1α), STAT3 and NF-κB, which in turn promotes the production of IL-6, IL-1β and TNF-α [89,90,91] (Figure 3). In addition, the ROS induced by macrophage activation upregulated the dimeric PKM2 and then attenuated the pro-inflammatory M1 macrophage phenotype while promoting the typical M2 macrophage characteristics [92]. A recent study found elevated levels of PKM2 in circulating and synovial fluid in RA patients and a positive correlation with RA disease activity [93]. These results suggest that PKM2 is a potential therapeutic target for RA.

ROS are known to maintain the homeostasis of macrophages, especially during macrophage polarization [85,94]. M1 and M2 macrophages have also been characterized as oxidative and reducing macrophages based on intracellular glutathione content [95]. In vitro studies have shown that ROS play an important role in the differentiation of macrophages into M2 based on the inhibition of ROS by butylated hydroxyanisole, and it has been concluded that M1 cells can develop without ROS, while M2 cannot [96]. Thus, given the essential role of MLSs in driving inflammation in the RA synovium, the deletion of inflammatory MLSs or the promotion of the repolarization of M1 macrophages to an anti-inflammatory M2 phenotype is a potential treatment for RA.

### 3.3. The Role of ROS in ECs

Angiogenesis is an early event in the pathogenesis of RA in which preexisting blood vessels promote the entry of blood-derived white blood cells into the sublining layer of the synovial membrane, thereby generating and enhancing inflammation [97]. Angiogenesis is associated with inflammatory cell infiltration and synovial tissue proliferation during RA disease progression. Macrophages and fibroblasts in the synovial tissue of RA respond to inflammatory stimulation by transforming the lining layer into a proliferative tumor-like “pannus”, damaging adjacent articular cartilage and bone [98,99]. In addition, various cells in the synovial membrane secrete a variety of angiogenic mediators and inhibitors, and the imbalance between them leads to the abnormal activation of endothelial cells (Ecs) and blood-derived endothelial progenitor cells [100]. Vascular endothelial growth factor (VEGF) is a potent angiogenic factor that induces EPC proliferation and migration, and facilitates angiogenesis, enabling the development of RA [101,102].

In RA, endothelial dysfunction as evidenced by an inflammatory response and impaired vasodilatory function, and a proclivity for thrombosis leads to accelerated atherosclerosis [103]. Increased ROS production, decreased endothelial NO synthase and increased NO consumption are all hallmarks of endothelial dysfunction [104]. Etanercept, a TNF inhibitor, reduces VEGF expression and production as well as NO and inducible NO synthase and improves endothelial function, lowering the risk of acute cardiovascular disease [105]. In addition, ROS can not only regulate VEGF, but can also play an important role in stabilizing the vascular system by regulating the intracellular signaling pathway of endothelial cells [106].

**Figure 4:**
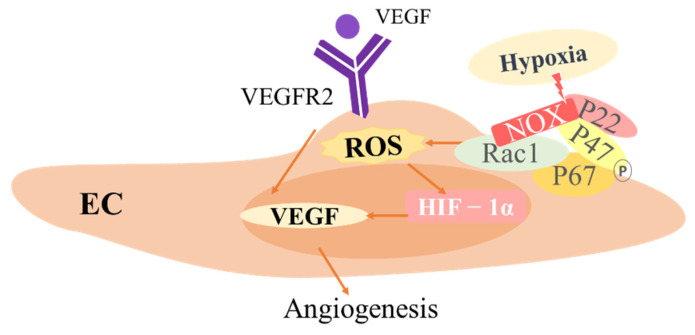
The role of ROS in endothelial cells (ECs). Vascular endothelial growth factor (VEGF) stimulates ROS production through RAC1-mediated activation of NOX, followed by the autophosphorylation of VEGFR2 and activation of downstream signaling pathways critical for endothelial cell migration and proliferation. In addition, hypoxia activates the transcription factor HIF-1α by inducing ROS production, which in turn upregulates VEGF secretion and expression and promotes angiogenesis.

Similar to MLS and FLS cells, Ecs also produce ROS, such as O_2_^•^^−^ and H_2_O_2_. NADPH oxidase is the most important source of ROS in the vasculature [107,108]. NADPH oxidase consists of membrane-bound gp91(phox) and p22hOx as well as cellular solute subunits, such as p47 (phox), p67 (phox) and the small GTPase Rac. In ECs, homologues of gp91(phox) (NOX2) are also expressed, including Nox1, Nox4 and Nox5 [109]. ROS derived from NADPH oxidase are involved in endothelial dysfunction and permeability, apoptosis and senescence, vascular cell growth and migration, remodeling, and inflammation, leading to various diseases, such as RA [109]. Conversely, endothelial migration and tubular structure were reduced after Nox-1 silencing by inhibiting PPARα, a regulator of NF-κB [110]. Rac1 protein, the regulatory subunit of the NOX family, is closely related to the recombination of F-actin fibers, which regulates the initial polarization of cells towards a predetermined direction of migration. VEGF stimulates ROS production through the RAC1-mediated activation of NOX, followed by the autophosphorylation of VEGFR2 and activation of downstream signaling pathways critical for endothelial cell migration and proliferation [111] (Figure 4). In addition, the non-phosphorylated form of p66Shc is associated with Rac1 in ECs. Endogenous consumption of p66Shc inhibits VEGF-induced Rac1 activity and ROS production and reduces the number of capillary branches, branching buds and tube length [112]. Monocyte chemotactic protein (MCP)-1 is a member of the pro-inflammatory CC chemokine superfamily that recruits monocytes and lymphocytes during the inflammatory response. TNF-α induces MCP-1 expression in endothelial cells through the ROS of RAC1-activated NADPH oxidase, which can be reversed by iron chelators and hydroxyl radical scavengers [113].

Hypoxia, TNF-α and interleukin (IL)-1 promote NOX expression in synoviocytes in the inflamed joint of a patient with RA [114], and persistent NOX-2 activation has been demonstrated to cause local oxidative stress [29]. In addition, the high expression of NOX-2 in synovial tissues is closely associated with markers of synovial vessels and synovitis, such as VEGF, ANG-2 and factor VIII [115]. Monika and colleagues found that the decrease in NOX2 expression was parallel to the increase in tPO2 levels in vivo only in patients who responded to TNFi treatment. In vitro Nox-2 activators (TNF-α and 4-HNE) and 3% hypoxia significantly promoted HMVEC migration, angiogenic tube formation and secretion of pro-angiogenic mediators, and these effects were blocked by NOX2 inhibition [115]. This information further reinforces that oxidation-driven angiogenesis may play a role in the pathogenesis of RA.

Sirtuin-1 (SIRT1) is a protein deacetylase that is activated by nicotinamide dinucleotide (NAD+). When cells are exposed to oxidative stress, FoxO1 interacts with SIRT1 to form a complex that activates cell cycle arrest/anti-stress genes, resulting in cell survival [116]. SIRT1 expression in RA ECs and synovial vasculature was reduced. Through the acetylation of p53 and P65, insufficient SIRT1 expression can enhance the proliferation and activation of endothelial cells as well as the development of angiogenesis [117]. Conversely, in an animal model of methyl-bovine serum albumin (mBSA)-induced arthritis, sirT1 activation reversed the pathogenic phenotype of RA ECs and alleviated the symptoms [117]. These findings corroborate SIRT1’s role in RA angiogenesis targeting and suggest that it could be employed as a supplementary therapy for RA [118].

HIF-1α is involved in the cellular response to hypoxia. HIF-1 does not undergo PHD-mediated hydroxylation during the quick transition from normoxic to hypoxic conditions; hence, it remains stable [119]. HIF-1 then activates its target gene VEGF, which promotes angiogenesis and causes synovitis [120]. Furthermore, the response of HIF-1α to hypoxia is related to hypoxia-induced ROS production [121]. According to Ying et al., hypoxia stimulates angiogenic tube formation and cell migration in HDMECs, which is accompanied by elevated G6PI and HIF-1 expression [122] (Figure 4). Melatonin (MLT) is a natural hormone that controls angiogenesis by reversing hypoxia/VEGF/H_2_O_2_-induced increases in cell survival and tube formation [123]. In addition, the combination of a HIF-1 inhibitor (KC7F2) and MLT inhibited HUVEC survival and tube formation by reducing the release of ROS and VEGF [124].

In summary, these findings show a molecular link between redox signaling and endothelial cell activities, implying that oxidative stress could be a catalyst for angiogenesis.

## 4. ROS and Synovial Immune Cells

### 4.1. The Role of ROS in Neutrophils

A large number of neutrophils with an abnormal, activated phenotype have been found in the synovial joints and tissues of RA patients, and they are characterized by increased production of ROS and cytokines, and delayed apoptosis [125,126]. Neutrophils recruited to the synovial environment guide the inflammatory response by phagocytosis and the release of granule enzymes and ROS, as well as the formation of a network called neutrophil extracellular traps (NETs), and they guide the innate and adaptive immune response by secreting cytokines and chemokines [127]. Myeloperoxidase (MPO) is the most abundant and most toxic enzyme found in neutrophil azurophil granules [128]. MPO and MPO-derived oxidants can not only promote inflammation and lead to tissue damage, but also increase endothelial dysfunction through the production of HCIO to induce vascular permeability and promote the development of RA [129]. In addition, neutrophils at the pannus-cartilage junction are also stimulated by immune complexes deposited locally. They engage in frustrated phagocytosis, releasing HOCl, ROS and cartilage-degrading enzymes into a microenvironment on the cartilage surface [130,131]. 

**Figure 5:**
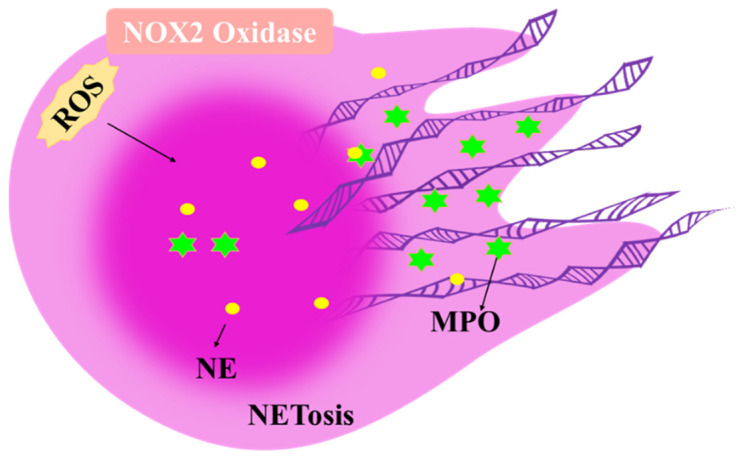
The role of ROS in neutrophils. ROS trigger the translocation of neutrophil elastase (NE) to the nucleus, where myeloperoxidase (MPO) then binds to chromatin and synergistically enhances chromatin deconcentration, leading to cell rupture and NET release.

NETs were identified in dying neutrophils as extracellular strands of decondensed DNA in association with histones and granule proteins [132]. NETs expose nuclear neoepitopes (e.g., citrullinated proteins) to the immune system, initiating the production of autoantibodies (e.g., ACPA) [133,134]. The formation of NETs requires the production of ROS [135]. NADPH oxidase mutations in neutrophils from patients with chronic granulomatous disease disrupt ROS production and fail to form NETs [136]. A burst of ROS triggers the translocation of neutrophil elastase (NE) to the nucleus, where MPO then binds to chromatin and synergistically enhances chromatin deconcentration, leading to cell rupture and NET release (Figure 5). This process can be blocked by the NADPH oxidase inhibitor diphenyliodonium (DPI) or the neutrophil-specific protease NE and protease 3 inhibitor (NEi) [137,138]. ROS generation is mediated by NADPH oxidase activation and mitochondrial release, both of which are essential for NETosis [139]. Thus, targeting NETs to reduce synovitis oxidative damage and restore immune tolerance may be a promising treatment option for RA. However, the degradation of neutrophil serine proteases (NSPs), the intrinsic pro-inflammatory mediator of NETs, inhibits aseptic inflammation in gout and SLE mouse models [140,141]. This technique could be part of the body’s overall strategy to break the neutrophil infection’s self-amplifying positive feedback loop. This action facilitates the resolution of the infection and acts as a protective measure against neutrophil infection.

Nevertheless, there is increasing evidence that NOX2-derived ROS play an important role in neutrophils as signal intermediates [142]. By phosphorylating NCF1, RA synovial neutrophils can display enhanced NADPH oxidase activity. This enzyme may emit O_2_^•^^−^, contributing to high levels of ROS, aggravating inflammatory processes and causing tissue damage [143]. Mutant Ncf1 caused a reduction in intracellular ROS, which resulted in more severe collagen-induced arthritis [144,145]. Furthermore, ROS play an important role in neutrophil chemotaxis. Sankarbarua et al. found that NOX2-dependent superoxide production was impaired in neutrophils in bone marrow cell 1 (TREM-1)-deficient mice. It was proposed that TREM-1 partially regulates neutrophil migration by promoting AKT activation and NOX2-dependent superoxide production [146]. Xie et al. found elevated levels of acute inflammatory markers, such as IL-1β, IL-6 and TNFα, in serum-induced joint inflammation under NOX2 deficiency, resulting in more severe arthritis. Treatment with an IL-1β blockade and cathepsin inhibitor was effective in Ncf1^−/−^ mice [147]. In addition, investigators found reduced PD-L1 expression on Ly6C^+^ neutrophils from arthritic tissues of Ncf1^−/−^ mice, and treatment with recombinant PD-L1-FC reduced arthritis severity and cytokine expression in Ncf1^−/−^ mice [148]. These results indicate that NOX2-derived ROS are essential for regulating the function and gene expression of RA neutrophils. Moreover, due to abnormal redox regulation, the anti-inflammatory function of NOX2-deficient neutrophils is weakened. Fernando et al. showed that H_2_O_2_ levels increased during neutrophil influx into synovial tissue, and increased H_2_O_2_ levels (endogenous or exogenous) inhibited p-Akt/NF-κB and induced the apoptosis of migrating neutrophils. It was suggested that H_2_O_2_ production is necessary for the spontaneous resolution of neutrophil inflammation [149]. Interestingly, the lack of NCF1-mediated ROS production did not alter collagenase-induced OA (CiOA) in arthritis [150] or reduce cartilage damage [151]. However, in a recent study, treating Ncf1-mutated animals with the NOS inhibitor L-NAME during the priming phase rather than the effector phase inhibited CII-induced arthritis [152].

The strong pro-inflammatory and diminished anti-inflammatory capabilities of neutrophils may be targets of treatment for immune-mediated arthritis due to aberrant redox regulation.

### 4.2. The Role of ROS in T Cells

T lymphocytes play an important role in the progression of RA and control the stability/progression of RA in the autoimmune stage [153]. Peripheral blood T cells in healthy HLA-DR4^+^ individuals and asymptomatic first-degree relatives of RA patients have typical telomere loss and abnormal T-cell differentiation, suggesting that the immune system is remodeling before disease [154]. Recent studies have shown that abnormal immune metabolism is closely related to ROS in CD4^+^ T cells, and that incorrect differentiation is an important mechanism of synovial inflammation in RA patients [115]. In RA, instead of transforming into memory and effector T cells, naive resting CD4^+^ T cells from lymphoid organs, such as lymph nodes and bone marrow, transform into short-lived effector T cells and rapidly enter the synovial tissue environment, where they undergo cell progression, triggering intense inflammation [153,155]. 

**Figure 6:**
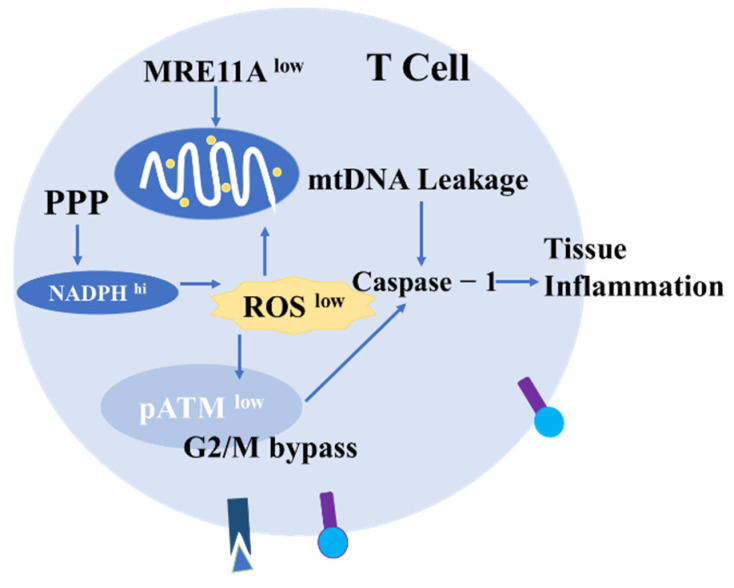
The role of ROS in T cells. In T cells, inadequate activation of the DNA repair kinase ATM bypasses the G2/M cell cycle checkpoint due to intracellular ROS consumption. In addition, MRE11A inhibition leads to reduced mitochondrial ATP and ROS production, mtDNA deposition, caspase-1 activation and invasive tissue inflammation.

It is known that 6-phosphofructo-2-kinase/fructose-2,6-bisphosphatase 3 (PFKFB3) is downregulated and 6-phosphoglucose dehydrogenase (G6PD) is upregulated in naive RA T cells. This change results in the conversion of T cells into the pentose phosphate metabolic pathway (PPP) [156,157]. With increased intracellular NADPH and ROS consumption [158], cellular redox signaling within T cells is impaired, the DNA repair kinase ATM is not activated, G2/M cell cycle checkpoints fail and T cells differentiate into imbalanced IFN-γ and Ll-17-producing inflammatory cells [91,157,159,160,161] (Figure 6). In addition, due to the excess NADPH and the decrease in ROS levels in the cells, RA T cells suffer from the dysregulation of adipogenesis, increased membrane shrinkage and the overexpression of the motor module dominated by the scaffold protein Tks5, leading to the rapid infiltration of T cells into synovial tissues [159,162,163]. Due to exposure to lactate-rich synovial fluid, these T cells are more likely to invade and remain in synovial tissue [154,163,164].

MRE11A is a DNA nuclease that functions as an exonuclease and endonuclease and is involved in single- and double-strand break repair [164,165]. Yinyin et al. found low MRE11A expression in RA-naive CD4^+^ T cells during the differentiation of invasive pro-inflammatory effector cells. Immunosenescence is characterized by adaptive immune failure and inadequate inflammatory suppression, and the inhibition of MRE11A activity in healthy T cells also induces an ageing phenotype. In addition, these actions also inhibited MRE11A expression in human synovial chimeric mice, leading to tissue invasiveness and pro-arthritic effects by T cells. The overexpression of MRE11A alleviates synovitis [166]. Their most recent study showed that the inhibition of MRE11A expression in T cells leads to mtDNA leakage, caspase-1 protein hydrolysis and invasive synovial tissue inflammation, and mitochondrial failure further reduces intracellular ROS production, thereby exacerbating redox signaling failure. In turn, MRE11A overexpression protects mtDNA from oxidative damage and leakage into the cytoplasm, as well as synovial tissue from inflammatory attack [153,166] (Figure 6). This observation that low intracellular ROS concentrations play an important role in driving aggressive tissue pro-inflammatory effector T cells.

The human synovia-SCID chimeric model can be used to study the close relationship between effector T-cell oxidation imbalance and pathogenic behavior in RA synovial tissue. In this model system, a human synovium was transplanted into immunocompromised mice, and the subsequent injection of CD45ro-PBMC cells from RA patients induced intense synovitis [161,167]. Buthionine sulfoximine (BSO), menadione and sulforaphane have been shown to be very effective at reducing tissue inflammation by restoring the redox balance of T cells through the replenishment of intracellular ROS levels [161], implying that ROS are directly involved in anti-inflammatory effects.

Furthermore, ROS generation is important for T-cell transcription and proliferation in response to TCR signals [168,169]. Regulatory T cells (Tregs) are an important subset of CD4^+^ T cells that maintain self-tolerance by secreting immunosuppressive/anti-inflammatory cytokines and expressing inhibitory receptors [170]. Tregs need to increase ROS and OXPHOS to achieve anti-inflammatory effects [171].

These findings establish ROS as the most important link between cellular metabolism and protective vs. auto-aggressive T-cell immunity. A potential technique for treating RA could involve therapeutic therapies that target ROS increases rather than ROS elimination. 

### 4.3. The Role of ROS in B Cells

Through local auto-antibody production and immune complex-mediated inflammatory responses, the role of B cells and B-cell effector pathways in synovitis is considered to be a central component of the pathogenesis of RA [172]. Synovial B-cell infiltration, on the other hand, is a highly variable occurrence that is found in up to 40% of patients, ranging from complete absence to dense distribution within organized infiltrates [173,174]. A recent study discovered a considerable increase in B-cell synovitis in patients with end-stage treatment-resistant RA. As a result, it is being investigated as a possible source of prognostic and predictive biomarkers in RA [175]. Furthermore, autoantibodies form in the synovium rather than in the peripheral blood and are class-switched during the progression of RA, implying that the local synovial environment plays an important role in the creation and maturation of autoantibody-producing B cells [176,177].

ROS may regulate lymphocyte activation and B-cell activity, making it one of the mechanisms that protects against the onset of autoimmune illness. Khmaladze and colleagues established a positive selection of self-reactive B cells using knock-in mice expressing a germline-encoded, CII-specific IgH (B10Q.ACB). This strong arthritic resistance is broken by introducing a mutation into the Ncf1 gene that causes ROS deficiency in Ncf1-mutated B10Q. In ACB mice, increased T-cell responses and intramolecular epitope dissemination are linked to disease development, although there are no somatic mutations in autoreactive B cells [178].

In addition, NETosis is increased as a result of neutrophil discharge in the synovial fluid of RA patients. Tissue-resident B cells within the ectopic lymphoid structure have also been found to produce high-affinity immune complexes targeting NET by binding to FcγRI and FcγRIIIa on neutrophil surfaces to promote the release of NETosis and degradation enzymes and ROS [179,180]. In turn, citrullination and/or NET production by neutrophils promote the production of B-cell autoantibodies, especially ACPA [181]. These findings imply that a self-reinforcing cycle can result in a severe autoimmune reaction.

At present, there are few studies on the effect of ROS on B cells in RA, and more studies are needed for verification.

## 5. Targeted ROS Therapy for RA

### 5.1. Antioxidant Therapy

In RA, there is an overabundance of ROS in synovial tissue and fluid, and ROS are tissue destroyers that maintain inflammation by damaging proteins, membranes and DNA [182]. In addition, the unifying theme in synovial-stromal cells and innate immune cells is excessive mitochondrial activity, providing pro-inflammatory ROS and energy to fuel tissue destruction of effector function [27,183]. 

ROS have long been thought to be the cause of diseases that can be treated by inhibiting them. In cases of prolonged activation and inflammation, limiting the effects of oxidants could be beneficial. As a result, much effort has gone into the creation of antioxidant medicines aimed at reducing ROS formation and scavenging ROS. These measures include: using precursors to increase glutathione; increasing antioxidant enzyme production, particularly through NRF2 activation; NOX inhibition; mitochondrial antioxidant defense; dietary antioxidants and inhibiting aberrant redox signals, among other things [184].

Since MLS is one of the major sources of ROS and pro-inflammatory factors [185], targeting ROS removal, reducing M1 and promoting M2 have been proposed as primary targets for reducing inflammation in this disease [80]. Several strategies, such as ceria-based nanotheranostics [186], manganese ferrite/ceria co-decorated nanoparticles [187] or silver nanoparticles [188], have been applied. However, these drugs indiscriminately target SMs and proinflammatory MLS. Therefore, future macrophage therapies will target different populations within the synovial membrane and provide a better understanding of its function and potential identification markers.

While promising outcomes have been observed n in some experimental systems, clinical trials have shown that these medications have either limited efficacy or unforeseen negative effects [140,184]. N-acetylcysteine (NAC) is one of the most studied antioxidant agents for therapeutic treatment. A recent clinical study showed that NAC did not improve RA disease activity [189]. NRF2 activators are being investigated as potential agents for increasing antioxidant capacity and alleviating disease. Some NRF2 activators, however, may operate within other signaling pathways and disturb associated biological processes in addition to activating NRF2 and triggering antioxidant enzymes [190,191].

### 5.2. Oxidant-Promoting Therapy

Contrary to popular belief, disease-promoting genetic polymorphisms work by reducing ROS production [192]. Holmdahl and colleagues revealed the immunomodulatory role of ROS in a variety of disease models. The ROS produced by NOX2 inhibit T-cell activation, and this effect is transferred to interacting T cells by antigen-presenting macrophages [193,194]. This finding suggests that ROS may have a beneficial effect on the prevention of autoimmune diseases. Furthermore, the production of NoX2-dependent ROS is important in innate immune function and antimicrobial responses. Therefore, further understanding of NOX2 activation pathways in specific cell types, as well as downstream signaling pathways, is crucial for the development of NOX2-inducing agents for treating autoimmune diseases. In RA, the pro-inflammatory effector functions of T cells are suppressed by replenishing ROS pools, by disrupting the synthesis of the ROS quencher glutathione or by blocking glucose shunt into the PPP [161].

However, current ROS modifying medicines are not specific to myeloid cells [194,195]. Their systemic use increases systemic ROS levels and may lead to oxidative stress, inflammation and tissue damage. Therefore, creating ROS amplifiers that are triggered in the presence of components specifically expressed in bone marrow cells reduces the negative effects of ROS therapy and allows it to be used to treat auto-immune diseases. In addition, if ROS concentrations can be fine-tuned, even in the low concentration range, it is possible to use the ROS activation signaling network for therapeutic purposes. Cell metabolism is also important for ROS formation and proliferation, and targeting metabolic interference of redox-sensitive signaling events is also a potential therapeutic approach.

Herbal medicines and their active constituents, as natural goods, are regarded as viable solutions for RA treatment due to their efficacy and low toxicity. In addition, growing evidence suggests that these medicines can inhibit RA by regulating the mitochondrial apoptotic pathway in FLSs. In addition, we reviewed drugs that induce intracellular ROS to improve RA, including traditional Chinese medicine components and chemical drugs. In FLSs, drugs stimulate the increase in intracellular ROS primarily through the following signaling pathways to promote apoptosis (Table 1 and Table 2): the mitochondrial-dependent apoptotic pathway, NF-κB-mediated apoptotic pathways, mitogen-activated protein kinase (MAPK)-mediated apoptotic pathway, endoplasmic reticulum stress (ERS)-mediated apoptotic pathway and PI3K-Akt-mediated apoptotic pathway. Some chemicals suppress inflammation by STAT3 signaling and AKT signaling in T cells (Table 1 and Table 2). Due to their efficacy and low toxicity, herbs and their active ingredients are considered viable options for treating RA. In addition, there is increasing evidence that these drugs can inhibit RA by regulating ROS levels. Therefore, supplementation with pro-oxidants is a promising approach for RA treatment.

## 6. Conclusions

In this review, we briefly described the role of ROS in RA synovial tissue and synovial fluid. The ROS and relevant molecular mechanisms in various cells related to the synovial microenvironment in RA were also discussed. Lastly, we presented the advantages and shortcomings of antioxidant therapy and pro-oxidant therapy targeting ROS. Although the role of ROS in RA is becoming increasingly attractive, there are still many research challenges and knowledge gaps.

During the early stages of RA development, ROS may play a beneficial preventive role, such as reducing the pro-inflammatory response of T cells and/or inhibiting antigen presentation by phagocytes. In contrast, high ROS levels are often observed in synovial and synovial fluid, as well as in FLSs, MLSs, endothelial cells and neutrophils during the chronic phase of RA, resulting in cell and tissue damage and possibly directly or indirectly sustaining disease progression. Therefore, given that ROS are important signal transduction molecules, both inside and outside cells, balancing ROS levels in both temporal and spatial environments is critical to maintaining synovial homeostasis in the presence of microbial or sterile stimuli. Furthermore, depending on the environmental and genetic context, a single mutation might activate a wide and complex collection of pathways. [193]. In the synovial microenvironment of RA, ROS play a significant role in regulating the organization of synovial cell networks and planning synovial cell specialization changes. These abnormal extracellular signals shape the behavior of pathogenic cells during the onset and progression of disease. It is difficult to monitor ROS in living cells in real time because they are small and short-lived. Progress in this field, however, is contingent on the investigation of complexes, including ROS sources and ROS targets. 

In recent years, due to the limited role of antioxidants and pro-oxidants in treating RA, increasing attention has been given to the important role of active ingredients from traditional Chinese medicines in treating RA. In addition to some of the traditional herbal ingredients we mentioned above, there are other herbal active ingredients that treat RA by improving antioxidant effects and regulating the inflammatory, such as anthocyanin [212], puerarin [213] and others. It is suggested that the traditional herbal ingredients may restore the imbalance of redox in and outside the cell by regulating ROS balance, so they will be promising treatment methods. 

## Figures and Tables

**Table 1:** Active ingredients and chemical components in traditional Chinese medicine that induce the ROS level in RA-related cells to improve RA.

Extracts/Monomers/Chemicals	Cells	Tissues/Animal Model	Dose/Concentration	Results	Potential Pathways	References
Hypericin photodynamic therapy	MH7A	/	0.25–4 μM	Induced apoptosis; increased intracellular ROS; cleaved caspase 9, increased PARP; decreased NF-κB.	Mitochondrial apoptosis pathway, the death receptor pathway, NF-κB pathways	[196]
Hempseed oil	MH7A	/	1–2%	Promoted apoptosis; Increased intracellular ROS; up-regulating CHOP, GRP94 and GRP78; activated PARP.	ER stress-mediated apoptosis	[197]
Brazilin	RAFLS	/	10, 25 μg/mL	Increased autophagosome, LC3-II, intracellular ROS; inhibited NF-κB, reduced IL-6, IL-8.	Autophagic, NF-κB pathways	[72]
Shikonin	RAFLS	AA	2, 2.5, 3 μM; 1, 2 mg/kg/day, i g	Increased ROS, LC3-II/LC3-I, apoptosis; decreased ATP, TNF-α, IL-6, IL-1β, IL-8, IL-17A, and IL-10; upregulating Bax, caspase 3; downregulating Bcl-2.	Mitochondrial apoptosis pathway and PI3K-AKT-mTOR pathways	[198]
Icariin	RAFLS	/	0.1, 0.5, 1, 2.5, 5 μM	Inhibited migration, proliferation; induced G2/M phase arrest, apoptosis; increased Bax, activated- caspase 3, cleaved-PARP, Cyt-C, ROS; decreased Bcl-2, p p65, IκBα; MCMP (Δψm).	G2/M phase arrest; mitochondrial apoptosis pathway and NF-κB pathway	[199]
Oridonin	RAFLS	/	5, 10, 25, 40 μM	Triggered cell apoptosis; increased caspase 3, caspase 9, PARP, Cyt-C, ROS; inhibited p ERK1/2, p JNK1/2, MCMP (Δψm).	Mitochondrial apoptosis pathway	[200]
α-Mangostin	MH7A/RAFLS	/	10–100 μM	Promoted apoptosis; increased Cyt-C, ROS, caspase 3, caspase 9, p ERK1/2. Decreased MCMP (Δψm).	Mitochondrial apoptosis pathway, ERK1/2 signaling pathway	[201,202]
Apigenin	RAFLS	/	100 μM	Induced apoptotic pathway; activated MAPK, ERK1/2, caspase 3, caspase 7; increased intracellular ROS.	ERK1/2 signaling pathway, apoptosis pathway	[203]
*Eupatorium japonicum* Thunb	RAFLS	/	37.5 μg/mL	Induced apoptosis, ATF4, CHOP; decreased NF-κB, p38, IL-1β, MMP-9.	ER stress-mediated apoptosis, NF-κB pathway	[204]
Cryptotanshinone	MH7A/RAFLS	/	5 μM	Increased ROS; downregulated Bcl 2, p Akt p STAT3; upregulated Bad, caspase 3, PARP, p p38, p c Jun N-terminal kinase.	Akt, MAPK, STAT3 pathways, mitochondrial apoptosis pathway	[205]
β-Elemene	RAFLS	/	10–200 μg/mL	Promoted apoptosis; decreased MCMP (Δψm); increased Cyt-C, ROS, caspase 9, caspase 3, p p38 MAPK.	Mitochondrial apoptosis pathway, MAPK pathway	[206]
1,7-Dihydroxy-3,4-dimethoxyxanthone	RAFLS	/	8.7, 17.4, 34.7 μM	Upregulated GADD45α, p-p38; increased apoptosis, ROS; inhibited NF-κB.	NF-κB/p38 pathway, apoptosis pathway	[207]
Scopoletin	rFLS (AIA)	/	250, 500, 1000 µM	Upregulated Bax, caspase 3; decreased MCMP (Δψm), Bcl-2, NF-κB.	Mitochondrial apoptosis pathway, NF-κB pathway	[208]
Resveratrol	RAFLS	/	40, 80, 160, 320 μM	Downregulated Bcl-2, Atg5, LC3B; increased ROS; released Ca^2+^.	Mitochondrial apoptosis pathway and autophagy	[73]
Sulforaphane	RA-T cell	/	0.5, 1, 2.5, 5, 10 μM	Inhibited CD25/CD69, proliferation; increased intracellular ROS, decreased GSH, p STAT3, RORγt, IL-17A, IL-17F, IL-22.	STAT3 signaling	[169]

**Table 2:** Chemical components that induced the ROS level of RA-related cells to improve RA.

Extracts/Monomers/Chemicals	Cells	Tissues/Animal Model	Dose/Concentration	Results	Potential Pathways	References
Suberoylanilide hydroxamic acid	RAFLS	/	0.5–10 μM	Induced apoptotic pathway; increased intracellular ROS, total IκBα; decreased p IκBα, NF-κB p65, Bcl-xL, Mcl-1.	Apoptosis pathway, NF-κB pathways	[209]
Niclosamide	RAFLS	/	0.25, 0.5, 1 μM	Induced apoptotic pathway; increased intracellular ROS, Bax, Cyt-C, caspase 9, caspase 3; Decreased p Akt, Bcl-2.	Mitochondrial apoptosis pathway, Akt pathways	[210]
Mitomycin C	RAFLS	/	10, 25, 50, 100 μg/mL	Induced apoptosis; increased intracellular ROS, Cyt-C, Bax/Bcl-2, caspase 9, caspase 3, PARP; decreased MCMP (Δψm).	Mitochondrial apoptosis pathway	[211]
Menadione	CD4 naive (CD4^+^CD45RO^−^) T cell	Human synovial tissue-NSG chimaera	3 μM; 10 mg/kg/day, i g	Increased ROS, p ATM, T-bet, RORγ; decreased IFN-γ, IL-17, TNF-α, IL-1β, IL-6, RANKL; inhibited spontaneous hypermobility.	ATM signaling	[161]
Buthionine sulfoximine	/	Human synovial tissue-NSG chimaera	1000 mg/kg/day, i g	Increased intracellular ROS; decreased IFN-γ, IL-17, TNF-α, IL-1β, IL-6, RANKL, GSH, T-bet, RORγ; inhibited spontaneous hypermobility	ATM signaling	[161]
